# ¿Carcinoma de mama o carcinoma de glándula sudorípara? Presentación de dos casos y análisis de la literatura

**DOI:** 10.7705/biomedica.5758

**Published:** 2021-09-22

**Authors:** Mauricio Luján, Gabriel Várela, Diego Morán

**Affiliations:** 1 Oncología Clínica, Clínica de Oncología Astorga, Medellín, Colombia Clínica de Oncología Astorga Medellín Colombia; 2 Facultad de Medicina, Universidad Pontificia Bolivariana, Medellín, Colombia Universidad Pontificia Bolivariana Facultad de Medicina Universidad Pontificia Bolivariana Medellín Colombia; 3 Patología Oncológica, Hospital Pablo Tobón Uribe, Medellín, Colombia Hospital Pablo Tobón Uribe Medellín Colombia; 4 Patología Oncológica, Clínica Aurora, Medellín, Colombia Clínica Aurora Medellín Colombia; 5 Patología Oncológica, Hospital San Vicente Fundación, Medellín, Colombia Hospital San Vicente Fundación Medellín Colombia

**Keywords:** neoplasias de la mama, neoplasias cutáneas, glándulas apocrinas, patología, informes de casos, Breast neoplasm, skin neoplasm, apocrine glands, pathology, case reports

## Abstract

El carcinoma apocrino primario de glándula sudorípara es una neoplasia con una muy baja incidencia, que puede representar un reto diagnóstico, clínico e histológico, y un reto terapéutico local, adyuvante y de la enfermedad avanzada. La edad media de los pacientes es de alrededor de 67 años, y no se ha observado preferencia según el sexo. Se presenta con mayor frecuencia en las axilas y en el cuero cabelludo. Se caracteriza clínicamente por un lento crecimiento, aunque puede progresar agresivamente, con compromiso local, ganglionar y metastásico, principalmente, pulmonar, hepático y óseo. El tratamiento recomendado -una vez establecida la histología- consiste en una resección local amplia con un margen claro de 1 a 2 cm y linfadenectomía regional si se detectan ganglios clínicamente positivos. El tratamiento adyuvante (radioterapia o quimioterapia) y de la enfermedad avanzada no está claramente establecido.

Se presentan dos pacientes de sexo femenino con sospecha inicial de cáncer de mama, en quienes se diagnosticó finalmente un carcinoma apocrino de glándula sudorípara.

## Abstract (en)

Primary apocrine carcinoma of the sweat gland is a neoplasm with a very low incidence that may represent a clinical and histological diagnostic challenge, as well as for adequate local, adjuvant, and advanced disease management. The average age of patients is around 67 years with no gender preference. This cancer develops primarily at the axillary and scalp levels and is clinically characterized by slow growth, but can progress aggressively with local, nodal, and metastatic involvement (primarily lung, liver, and bone). The recommended management, once the histology is established, consists of a wide local resection with a clear margin of 1 to 2 cm and regional lymphadenectomy if clinically positive nodes are detected. The adjuvant treatment (radiotherapy or chemotherapy) and for the advanced disease is not established.

We report here the cases of two female patients initially diagnosed with breast cancer who were finally diagnosed with apocrine carcinoma of the sweat gland.

El carcinoma apocrino primario de glándulas sudoríparas es una neoplasia extremadamente rara con pocos casos reportados en la literatura científica. La incidencia es bastante baja: de 0,0049 a 0,0173 casos por 100.000 personas al año.

Se caracteriza clínicamente por un lento crecimiento, lo que lo hace confundir con un tumor benigno lo cual puede retrasar el diagnóstico; no obstante, puede progresar agresivamente y producir compromiso local, ganglionar y metastásico. La edad media de presentación de los pacientes es de alrededor de 67 años y no se ha observado preferencia según el sexo. El cáncer se desarrolla principalmente en las axilas y el cuero cabelludo, aunque también se ha reportado en diversos sitios anatómicos [cabeza y cuello (cara, canales auditivos, párpados), tronco, extremidades y área anogenital].

El tratamiento recomendado -una vez establecida la histología- consiste en una resección local amplia con un margen claro de 1 a 2 cm y linfadenectomía regional si se detectan ganglios clínicamente positivos. El tema más controvertido es el tratamiento adyuvante (radioterapia o quimioterapia). Estos tumores pueden recurrir localmente y en los ganglios, e inclusive, producir metástasis sistémicas en pulmón, hígado y huesos. Dada su baja incidencia, puede representar un reto diagnóstico, clínico e histológico, y un reto terapéutico, local, adyuvante y de la enfermedad avanzada ^1^.

Se presentan dos casos de carcinoma apocrino primario de glándulas sudoríparas, ambos en mujeres, uno en axila, inicialmente manejado como posible cáncer primario de mama, y uno en la piel cervical posterior, inicialmente informado por patología como metástasis de cáncer de mama.

## Caso clínico 1

Se trata de una mujer de 69 años, valorada por el Servicio de Oncología en junio de 2020, con antecedentes de tiroidectomía total por carcinoma papilar de tiroides en estadio I a los 62 años. Recibió yodo radiactivo y posterior suplencia hormonal con levotiroxina. Como antecedentes familiares, había tres hermanas con cáncer de mama y, la madre y dos hermanos, con cáncer gástrico. No había otros datos de importancia y la paciente negó contacto con tóxicos. En cuanto a los antecedentes ginecoobstétricos, había tenido tres embarazos, tres partos y ningún aborto; la menarquia ocurrió a los 13 años y, la menopausia, a los 50.

Fue remitida inicialmente a Dermatología, y refirió presentar un nódulo eritematoso de crecimiento progresivo desde abril del 2019, en la piel de la región lateral izquierda occipito-cervical. En una ecografía de junio de 2019, se reportó una lesión nodular hipoecoica, bien definida, avascular, de 14 x 9 mm, sugestiva de ser un ganglio. Se practicó una biopsia por aspiración con aguja fina en junio de 2019, cuyo resultado fue negativo para neoplasia. Ante un leve crecimiento, se decidió hacer una biopsia por escisión en octubre de 2019, en la que se reportó un compromiso intradérmico por tumor epitelial.

Los estudios de inmunohistoquímica fueron positivos para GATA3, CK20, receptores de estrógenos y progestágenos y CK7, y negativos, para TTF1 y HER2. Con un KI67 de 60 %, se sugirió el diagnóstico de carcinoma infiltrante de grado 2 de origen mamario. Los exámenes practicados -endoscopia digestiva superior, colonoscopia, ecografía transvaginal, tomografía de tórax y abdomen, y citología vaginal- fueron normales. La mamografía de noviembre de 2019 fue reportada como BI-RADS 2.

**Figura 1:**
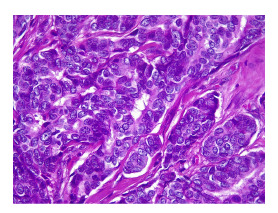
En la microfotografía se aprecia una neoplasia constituida por cordones estrechos de células epiteliales displásicas con citoplasma anfófilo que adoptan un patrón expansivo invadiendo el estroma. Nótese la leve atipia nuclear. Hematoxilina y eosina, 40X.

**Figura 2:**
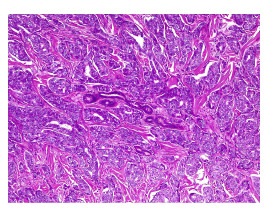
Se observa la distribución irregular de los nidos entre el estroma colagenizado, esbozando áreas sólidas y cribiformes sin necrosis. En el centro se visualiza un ovillo de glándulas sudoríparas que están siendo rodeadas por el tumor. Hematoxilina y eosina, 10X.

**Figura 3:**
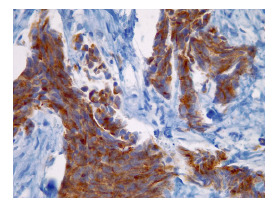
Cromogranina (inmuhistoquímica), 40X.

**Figura 4:**
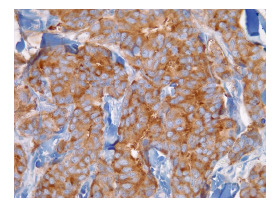
Sinaptofisina (inmunohistoquímica), 40X.

Con estos estudios fue remitida al Servicio de Oncología Clínica donde en el examen físico se documentó cicatriz en la región cervico-occipital lateral izquierda, con leve fibrosis, sin lesiones tumorales palpables o visibles en piel, tejidos blandos, cavidad oral, cervical, axilar o mamario. Se solicitó una revisión de la histopatología, y se hizo un nuevo panel de inmunohistoquímica con reporte positivo para sinaptofisina, cromogranina, BER-EP4, GATA3 y andrógenos, y negativo para CD56, CK5, CK6, CK20, D240, PAX8, tiroglobulina y TTF1. Con base en estos resultados, se consideró que se trataba de un carcinoma de glándula sudorípara con diferenciación neuroendocrina y mucinosa (figuras 1-4).

## Caso clínico 2

Se trata de una mujer de 70 años valorada en el Servicio de Mastología el 10 diciembre del 2019. La paciente tenía antecedentes personales de hipertensión arterial sistémica y dislipidemia controladas, pero sin otros antecedentes familiares o tóxicos de importancia.

Reportó una lesión nodular en la mama derecha en agosto de 2019. En la mamografía de ese momento, se reportó una categoría BI-RADS 4b por la presencia de una masa densa de 26 mm en la unión de los cuadrantes externos y el ganglio axilar derecho. En una ecografía del 18 de septiembre del 2019, se reportó una lesión nodular de 15 x 11 x 7 mm en la unión de los cuadrantes externos y el ganglio axilar derecho de 15 mm. La biopsia con aguja *Trucut* guiada por ecografía del 23 de agosto del 2019 no evidenció neoplasia maligna ni atipias, solo necrosis. Se ordenó una nueva biopsia de mama y de ganglio axilar el 20 de septiembre del 2019, en la cual no se evidenció neoplasia maligna de mama, pero sí focos de adenosis esclerosante y ganglio axilar positivo para neoplasia maligna.

En la resonancia magnética de mamas del 30 de octubre del 2019, se detectó un patrón de captación del tipo de masa derecha irregular, con un tamaño de 13 x 8,7 x 8,1 mm y realce heterogéneo en la fase inicial rápida y posterior de meseta (tipo 2) superior y medial a esta, así como un área de realce del tipo 'no masa' de 24 x 8 x 23 mm con realce heterogéneo en la fase inicial rápida y posterior de meseta (tipo 2) más restricción de difusión y ganglios axilares en las cadenas 1, 2 y 3 de hasta 7,7 mm. No se observaron ganglios mamarios internos; la mama izquierda aparecía normal.

En una nueva biopsia de mama el 15 de noviembre del 2020, no se evidenció neoplasia maligna. En el examen físico no se detectaron hallazgos mamarios claros, pero se palparon adenomegalias móviles de 25 mm en la axila derecha. Se decidió hacer una biopsia abierta de ganglio axilar el 8 de enero del 2020, en la cual se encontró compromiso por neoplasia de célula grande de patrón difuso, sin estructuras tubulares y tejido axilar mamario supernumerario sin malignidad. En la prueba de inmunohistoquímica, se reportaron resultados positivos para CK, S100, CK7, SOX10 y p53; y negativos, para CK20, GATA3, TTF1, p63 y melan-A, ACL, así como para receptores hormonales, con un Ki67 de 15 % y HER2 negativo.

En marzo del 2020, en una tomografía por emisión de positrones-tomografía computarizada (PET-TC), se evidenciaron lesiones hipercaptantes en la axila derecha, la región supraclavicular y la infraclavicular y la base del cuello en el lado derecho, resultados que se interpretaron como un posible tumor primario de mama TxN3M0.

Se decidió hacer mastectomía más vaciamiento ganglionar en abril del 2020, cuya histopatología no registró actividad tumoral en el tejido mamario; no obstante, hubo 5 ganglios positivos de 15, y nidos neoplásicos similares a los reportados en estudios previos de histopatologías. Una vez valorada en el Servicio de Oncología Clínica, en abril del 2020 se decidió iniciar quimioterapia adyuvante con un esquema de doxorrubicina y ciclofosfamida en dosis densa.

**Figura 5:**
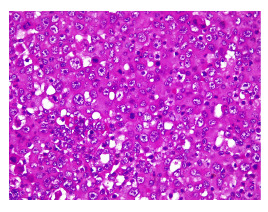
La imagen histológica muestra un tumor formado por células grandes, núcleos hipercromáticos, nucléolos prominentes y múltiples figuras mitóticas. El citoplasma es abundante e intensamente eosinófilo. Hematoxilina y eosina, 40X.

**Figura 6:**
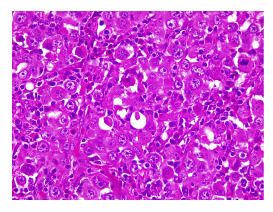
Nótese la formación glandular y la presencia de secreción granular eosinofílica intraluminal proveniente de la decapitación de la célula tumoral. Varias células presentan gránulos finos citoplasmáticos. Los linfocitos maduros que rodean y penetran la neoplasia corresponden a la localización ganglionar del tumor. Hematoxilina y eosina, 40X.

**Figura 7:**
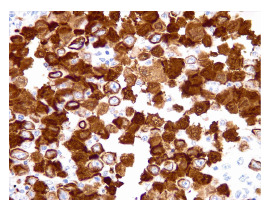
Citoqueratina 7 (inmunohistoquímica), 40X.

**Figura 8:**
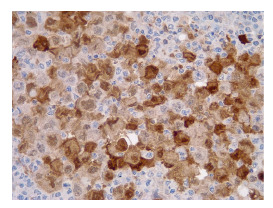
S100, 40X

Al hacer una nueva revisión de todo el material de histopatología y los estudios de inmunohistoquímica, se reportaron resultados negativos para HMB45, CD30, CD5, HLA-DR, CD43, CD87, CD7, CD4, CD2, CD1A, melan-A, CD45, CD20, ALK1, p63, PAX5, CD3, CD56, GATA3, andrógenos y PLQ, y positivos para EMA, CK, SOX10, CK7 y S100, por lo que se consideró que se trataba de un adenocarcinoma apocrino de glándula sudorípara primario axilar (figuras 5-8).

Se decidió suspender la quimioterapia iniciada y hacer una nueva PET-TC en junio del 2020 cuyo resultado fue negativo, por lo que se administró radioterapia adyuvante y se continuó el seguimiento clínico.

## Consideraciones éticas

Se observaron las normas éticas para la investigación en seres humanos contenidas en la Resolución 008430 de 1993 del Ministerio de Salud de Colombia y se mantuvo la confidencialidad de la información de las pacientes. Las pacientes autorizaron el uso de sus historias clínicas.

## Discusión

Los tumores de glándula sudorípara son tumores poco frecuentes. En un análisis de la base de datos del programa de *The Surveillance, Epidemiology, and End Results* (SEER) entre 1973 y 2013, se encontraron 5.298 pacientes con tumores de anexos localizados en cabeza y cuello, de los cuales 295 correspondían a tumores de glándulas sudoríparas y 104 a adenocarcinoma apocrino. La edad media en el momento del diagnóstico fue de 67 años. La incidencia calculada en Estados Unidos (base de datos de SEER) es de 0,0049 a 0,0173 por 100.000 habitantes por año. En el caso de lesiones de origen mamario, esta corresponde a menos del 1 % de los tumores triple negativos, lo mismo sucede con los tumores cutáneos, en los que los tumores benignos y malignos de anexos representan menos del 1 % de los tumores de piel no melanoma y se derivan de células ecrinas, apocrinas, sebáceas o ceruminosas de la piel o de los folículos ^2^.

El origen más probable de estas neoplasias es el tejido normal o modificado de células apocrinas y, aunque su etiopatogenia no ha sido aún bien descrita, al parecer la exposición a la luz ultravioleta puede ser un factor de riesgo. Desde el punto de vista molecular, se han descrito mutaciones en los genes supresores *PTCH1* y *CDH1*, y mutaciones somáticas en los genes *TCF7L1, ARID1A, FBX011, FNBP1, 1L6ST, MYC* y *WHSC1L*^3^.

Las neoplasias malignas de glándula sudorípara se han estratificado según su potencial de recurrencia y metástasis en dos grupos ^4^ (cuadro 1). El diagnóstico ha evolucionado con el tiempo y el uso de las técnicas de inmunohistoquímica ha dado mayor claridad, especialmente en el uso de anticuerpos como el EKH5 y el EKH6, que son específicos para los órganos con secreción ecrina ^5^. Otros criterios del diagnóstico histológico incluyen el material positivo para PAS y resistente a diastasa en células de la luz, y con positividad inmunohistoquímica para la GCDFP-15 ^6^, así como expresión positiva para el receptor de andrógenos. Es de resaltar que la expresión positiva del receptor de estrógeno puede estar presente hasta en el 36 % de los tumores apocrinos de glándulas sudoríparas ^7^. Para que puedan considerarse carcinomas apocrinos puros de mama deben tener un perfil negativo para el receptor de estrógenos y positivo para andrógenos, aunque esta diferencia puede ser ante todo académica porque su comportamiento es similar al de los *apocrine-like* en cuanto a la supervivencia global y libre de enfermedad ^8^. El uso del antígeno carcinoembrionario (CEA) y de los receptores del factor de crecimiento epidérmico (EGF) puede ser particularmente útil para diferenciar estos tumores de una metástasis de carcinoma mamario ^3^.

**Cuadro 1:** Clasificación del riesgo de las neoplasias malignas de glándula sudorípara ^4^

Bajo riesgo	Alto riesgo
Carcinoma cribiforme	Porocarcinoma
Carcinoma secretorio	Hidradenocarcinoma
Carcinoma endocrino de glándula sudorípara con producción mucinosa	Adenocarcinoma digital papilar
Carcinoma mucinoso	Carcinoma apocrino
Carcinoma microquístico anexial	Siringocistoadenocarcinoma papilífero
Carcinoma ductal ecrino escamoide	Neoplasias malignas originadas en espiroadenoma, cilindroma, espiradenocilindroma; con alto grado morfológico
Carcinoma adenoide quístico	Carcinoma histiocistoide
Neoplasias malignas originadas en espiroadenoma, cilindroma, espiradenocilindroma con bajo grado morfológico	Adenocarcinoma anexial NOS

NOS: *Not otherwise specified*

El carcinoma de glándula sudorípara endocrino productor de mucina es una neoplasia neuroendocrina usualmente de bajo grado, considerada por muchos autores como el análogo cutáneo del adenocarcinoma papilar sólido de glándula mamaria. Aunque en los primeros estudios se reportaba una predilección por el párpado y la piel periorbitaria, se ha encontrado en muchas otras localizaciones extraoculares y es un reto diagnóstico para los patólogos. En algunas ocasiones puede ser la lesión precursora de un carcinoma mucinoso cutáneo, por lo cual es importante conocer esta entidad recientemente descrita y reconocida en la última clasificación de tumores cutáneos por la Organización Mundial de la Salud (OMS) ^9^.

Histológicamente puede tener varios patrones, desde nódulos expansivos, quistes y papilas, hasta áreas sólidas y cribiformes. Las células son redondas a poligonales, tienen un tamaño intermedio y presentan una escasa tasa de mitosis. Usualmente no se observa necrosis. La cantidad de mucina intracelular o extracelular varía según el tumor y tiende a ser muy poco notoria en las fases iniciales o en las zonas sólidas.

El diagnóstico ha evolucionado con el tiempo y el uso de las técnicas de inmunohistoquímica ha significado una mayor claridad, utilizando un panel amplio para lograr identificar su histogénesis, ya que a veces es imposible diferenciarlo histológicamente de una metástasis de adenocarcinoma de mama. Los marcadores que suelen ser positivos (mas no específicos) son el CK7, CK8, CK18, AE1/AE3, CAM5.2, EMA, GCDFP-15, estrógenos y progesterona. Lo más característico es la expresión de marcadores neuroendocrinos como la sinaptofisina y la cromogranina ^10^.

En cuanto a su diagnóstico diferencial desde el punto de vista microscópico, debe considerarse el hidradenoma, el hidrocistoma con hiperplasia ductal papilar y el adenoma apocrino. Todos ellos son relativamente fáciles de diferenciar debido a la ausencia de componente infiltrativo, su menor tamaño y la ausencia de atipia. El principal reto diagnóstico es discriminarlo de una metástasis de adenocarcinoma papilar sólido de mama, pues pueden ser exactamente iguales, incluso en la inmunohistoquímica, siendo la correlación clínico-patológica el criterio de referencia. Es de resaltar que la expresión positiva del receptor de estrógeno puede estar presente hasta en el 36 % de los tumores apocrinos de glándulas sudoríparas ^7^.

El adenocarcinoma apocrino de glándula sudorípara es una inusual neoplasia cutánea de anexos, que se manifiesta histológicamente con signos inequívocos de secreción por decapitación o gránulos de cimógenos citoplasmáticos. Además, no debe detectarse a nivel microscópico ninguna otra lesión con diferenciación apocrina, como en el caso de siringocistadenocarcinoma papilífero, carcinoma mucinoso, carcinoma papilar digital o neoplasias benignas como cilindroma, espiradenoma y tumor mixto apocrino. Tampoco debe estar asociado u originado en una glándula especializada: las de Moll, las ceruminosas, las anogenitales y las de tipo mamario) ^11^.

Histológicamente, el tumor está conformado por células epiteliales con abundante citoplasma granular eosinófilo, parcialmente vacuolado. Puede tener diferenciación ductal y secreción por decapitación. El pleomorfismo y las figuras mitóticas son variables. El nucléolo tiende a ser conspicuo. En algunas ocasiones puede adoptar patrones morfológicos con células en anillo de sello o aspecto histiocitoide, similar al carcinoma de células en anillo de sello o histiocitoide periorbitario. En la inmunohistoquímica, las células tumorales son positivas para citoqueratina 7 y tienen expresión variable para CEA, S100, EMA, estrógenos y progesterona ^12^. Anticuerpos como el EKH5 y el EKH6 son específicos para los órganos con secreción ecrina y han demostrado ser útiles ^5^. Otros criterios para el diagnóstico histológico incluyen material positivo para PAS y resistente a la diastasa en células luminales, positividad inmunohistoquímica para GCDFP-15 ^6^, así como expresión positiva para el receptor de andrógenos.

El diagnóstico histopatológico diferencial se establece básicamente con la metástasis cutánea del adenocarcinoma ductal mamario, más aún si este tiene una morfología apocrina, situación que es difícil de sortear, inclusive teniendo la ayuda de la inmunohistoquímica, ya que ambos tumores pueden compartir el mismo perfil inmunológico y solo la ausencia de lesión mamaria puede discriminarlos. Hay algunos datos que pueden ser de ayuda para lograr diferenciarlos y determinar su origen, como la presencia de neoplasia *in situ* o evidencia de transición de una glándula apocrina preexistente. La expresión de EGFR (HER1), D2-40 y P63 puede apoyar más el diagnóstico del carcinoma primario que el del metastásico ^13^; sin embargo, cuando la enfermedad se presenta con compromiso axilar, la investigación clínica y la correlación patológica son lo más recomendable.

Clínicamente, estos tumores se caracterizan por ser masas sólidas o quísticas asociadas con cambios inflamatorios de la piel suprayacente y, en ocasiones, ulceración. Los sitios más frecuentes son la axila y la región anogenital, pero básicamente pueden aparecer en cualquier lugar donde haya glándulas con actividad apocrina. El patrón predominante de la enfermedad es localmente invasor, pero tiene habilidad para diseminarse sistémicamente, especialmente por vía linfática. En el 57 % de los casos, la enfermedad se diagnostica en un estadio localizado y, en un 5 %, en estadio metastásico; sin embargo, este porcentaje podría ser mayor si se usa PET-FDG como método de diagnóstico ^14^.

Como en los tumores cutáneos, el principal tratamiento es el quirúrgico, con resecciones radicales, con un margen libre de 1 a 2 cm. El papel de la disección ganglionar aún es debatido, aunque los análisis retrospectivos han demostrado que los pacientes con compromiso ganglionar tienen una menor mediana de supervivencia, 33 meses *Vs.* 55 meses cuando no hay compromiso ganglionar. A pesar de estos datos, hasta ahora el compromiso ganglionar se ha considerado como un factor de pronóstico, pues no existe una recomendación estándar en cuanto a su manejo y se desconoce si la disección ganglionar extensa puede modificar el curso de la enfermedad ^15^. El uso de las imágenes de resonancia magnética podría ayudar a planear de forma más precisa su tratamiento ^1^.

A diferencia de otras localizaciones, los tumores en cabeza y cuello se tratan más frecuentemente con disecciones ganglionares cervicales con o sin radioterapia cuando se ha determinado clínicamente o mediante ganglio centinela el compromiso ganglionar. Cabe resaltar que en esta localización hay una gran probabilidad de enfermedad metastásica, asociada principalmente a un mayor volumen tumoral en el momento del diagnóstico ^16^. En un análisis retrospectivo sobre tumores de anexos cutáneos localizados en cabeza y cuello, se encontró que la calidad de la resección se relacionaba directamente con el pronóstico, en tanto que otras variables, como la edad avanzada, el alto grado o el gran volumen tumoral, así como la presencia de compromiso ganglionar, se comportaban como factores de pronóstico adverso. La presencia de adenopatías se considera el principal factor pronóstico adverso para la supervivencia (HR=3,223; IC_9_5% 1,52-6,835; p=0,002) ^2^.

La radioterapia se ha indicado en los pacientes con enfermedad localmente avanzada, especialmente en quienes el margen es menor de 5 mm o es positivo, o que tienen un alto grado histológico o adenopatía ^6^. Además, se ha propuesto la radioterapia en los pacientes con tumores mayores de 5 cm moderadamente diferenciados o con invasión vascular y perineural ^17^. El papel de la quimioterapia parece limitarse a la enfermedad metastásica o recurrente sin posibilidades de manejo local ^18^. De hecho, en las evaluaciones de tumores apocrinos de la mama sin enfermedad metastásica, se ha encontrado que la administración de terapia adyuvante puede comportarse como un factor de pronóstico adverso (HR=4,41; IC95% 1,0119,19; p=0,048) ^19^. Se ha recomendado el uso de bleomicina, 5-fluorouracilo, carboplatino, metrotexato, paclitaxel, epirrubicina o ciclofosfamida, pero este carece de una base sólida ^1^^,^^20^^,^^21^. Según algunas experiencias anecdóticas en tumores con expresión del receptor de estrógenos, el uso de tamoxifeno ha dado resultados positivos ^22^^,^^23^. Los estudios de genética realizados en ciertos subtipos tumorales, como el carcinoma secretorio de piel, han permitido encontrar translocaciones 12;15, lo cual resulta en el gen de fusión *ETV6-NTRK3,* que podría ser objeto de intervención mediante los inhibidores del *NTRK,* pero su uso se basa en la racionalidad biológica más que en la evidencia clínica, tal como sucede con el adenocarcinoma papilar digital y la sobreexpresión de *FGFR2* como blanco terapéutico ^4^. En la práctica clínica, se administran medicamentos diseñados contra las mutaciones de *PTCH1* (vismodegib) y *TCF7L1*, pero su uso tampoco es extenso ^3^.

Estos tumores parecen tener un pronóstico diferente que sus contrapartes dependiendo de la localización de la enfermedad: en el caso de los tumores localizados en cabeza y cuello. la supervivencia global a 10 años es del 54 %, y la supervivencia libre de progresión, del 97 %, siendo más baja que en el caso de los tumores de piel no melanoma ^2^.

En el análisis de la literatura (búsqueda en Pubmed y Embase de artículos publicados e indexados entre 1975 y 2020, disponibles en texto completo en inglés, español, francés y alemán; no se consideraron comunicaciones orales o resúmenes; las palabras clave incluyeron los términos "glándulas sudoríparas" "carcinoma" "neoplasias cutáneas" "glándulas apocrinas"), se encontraron 102 casos de carcinoma apocrino primario de glándulas sudoríparas reportados entre 1978 y 2020 que, junto con los dos informados aquí, se resumen en el cuadro 2 ^1^^,^^3^^,^^15^^,^^16^^,^^18^^,^^24^.

**Cuadro 2:** Características clínicas de 104 pacientes con carcinoma apocrino primario de glándula sudorípara, reportados entre 1978 y 2020 ^1^^,^^3^^,^^15^^,^^16^^,^^18^^,^^24^

n=104 (100 %)	
Sexo	53 mujeres (50,9 %)
Edad media en años [DE]	63 [13]
Localización	
Axilar	36 (34,6)
Cuero cabelludo	25 (24,0)
Miembros inferiores	11 (10,5)
Miembros superiores	10 (9,6)
Tronco	4 (3,8)
Vulva	3 (2,8)
Cabeza y cuello	3 (2,8)
Perianal	1 (0,9)
Manejo local	
Escisión	54 (51,9)
Resección local amplia	26 (25,4)
Resección local amplia más vaciamiento ganglionar	17 (16,3)
No reportado	6 (5,8)
Manejo adyuvante	
Radioterapia	3 (2,9)
Radioterapia más quimioterapia	3 (2,9)
Quimioterapia	1 (0,9)
Recurrencia local o ganglionar	20 (19,2)
Metástasis	
En el momento del diagnóstico	1 (0,9)
Durante el seguimiento	9 (8,6)
Sitios de metástasis	
Ósea	5 (4,8)
Pulmón	5 (4,8)
Hígado	1 (0,9)
Cerebro	1 (0,9)
Mortalidad	15 (14,4)

Los carcinomas apocrinos primarios de glándulas sudoríparas son tumores infrecuentes para los cuales no se han establecido aún criterios diagnósticos y de tratamiento. La integración de los datos de histopatología y la presentación clínica son de gran importancia, ya que, particularmente en la axila (sitio más frecuente), se pueden afectar el tratamiento, el pronóstico y la calidad de vida del paciente. El manejo local adecuado y el compromiso ganglionar son los dos principales factores pronósticos y establecen la posible necesidad de tratamientos complementarios en torno a los cuales no hay claridad hasta la fecha.
